# Effect of ripasudil on diabetic macular edema

**DOI:** 10.1038/s41598-019-40194-5

**Published:** 2019-03-06

**Authors:** Yoshiro Minami, Young-seok Song, Akihiro Ishibazawa, Tsuneaki Omae, Tomoko Ro-mase, Satoshi ishiko, Akitoshi Yoshida

**Affiliations:** 1 Department of Ophthalmology, Nayoro City General Hospital, Nayoro, Japan; 2 Department of Ophthalmology, Asahikawa Medical University, Asahikawa, Japan

## Abstract

The current study aimed to address whether ripasudil, a Rho-associated coiled-coil containing protein kinase (ROCK) inhibitor developed to treat glaucoma and ocular hypertension (OH), improves diabetic macular edema (DME) since it is known that ROCK upregulates vascular endothelial growth factor. We retrospectively investigated the foveal thickness (FT) measured by spectral-domain optical coherence tomography, visual acuity (VA), and intraocular pressure (IOP) in 12 eyes with DME that received ripasudil treatment for primary open-angle glaucoma or OH and compared them with 14 eyes that received no treatment. One month after ripasudil therapy, the mean FT decreased significantly from 439 ± 72 µm to 395 ± 62 µm (*P* = 0.003); this change was significantly different from that in the controls, in which the mean FT increased by 1 ± 39 µm (*P* = 0.01). Ripasudil also caused a significant decrease in IOP from 17.3 ± 5.2 mmHg to 14.6 ± 4.0 mmHg (*P* = 0.02); this change was significantly greater than that in the controls, in which IOP changed by 0.0 ± 1.6 mmHg (*P* < 0.008). There was no significant difference in the VA changes between groups. Our results suggested that ripasudil may have positive effects on both IOP and DME.

## Introduction

Diabetic macular edema (DME) is the major cause of visual loss in patients who are of working age^1^. A recent meta-analysis that included 22,896 patients with diabetes showed that the prevalence of DME was 6.81%^2^. Although the pathogenesis of DME is multifactorial, i.e., capillary endothelial vascular dysfunction, local inflammatory activity, hypoxia, oxidative stress, breakdown of the blood-retinal barrier, and retinal neurodegeneration, vascular endothelial growth factor (VEGF) is a key player in the process^3^. Recently, intravitreal injections of anti-VEGF drugs have been gaining in popularity for treating DME, and many studies have investigated the effects of anti-VEGF drugs on DME^4–8^.

Ripasudil (Glanatec Ophthalmic Solution 0.4%, Kowa Company, Ltd., Japan) is the first ophthalmic solution using a Rho-associated coiled-coil containing protein kinase (ROCK) inhibitor developed to treat glaucoma and ocular hypertension (OH) in Japan^9–11^. ROCK signaling upregulates VEGF in diabetic retinopathy (DR) and plays important roles in the pathogenesis of microvascular complications in DR^12–14^. Previous studies have reported that a combination therapy of bevacizumab (Avastin, Genentech, Inc., South San Francisco, CA) and a ROCK inhibitor (fasudil) intravitreal injection was effective in eyes with severe DME that were refractory to anti-VEGF therapy^15,16^. In the current study, we showed the effect of ripasudil on DME in patients who also had glaucoma or OH.

## Materials and Methods

### Subjects

The ethics committee of our institution approved the study, which adhered to the tenets of the Declaration of Helsinki, and written informed consent was obtained from all study participants. The medical records of patients who visited Nayoro City General Hospital (Nayoro, Hokkaido, Japan) between January 2016 and December 2017 were reviewed retrospectively. The inclusion criteria were administration of ripasudil for primary open-angle glaucoma (POAG) (including normal-tension glaucoma) or OH (intraocular pressure [IOP] >21 mmHg), presence of DME before the administration of ripasudil (foveal thickness [FT] ≧300 µm), no history of ophthalmic drug changes except for ripasudil, no history of ocular surgery (including laser surgery), and/or no treatment for macular edema such as an intravitreal anti-VEGF drug injection within the previous 3 months and after the prescription of ripasudil until the first follow-up visit (2 to 8 weeks). No patients received a dexamethasone implant (Ozurdex, Allergan, Irvine, CA), which has not been approved in Japan. We enrolled 12 eyes from 10 patients in this study (ripasudil group). We also enrolled control patients based on the medical records of patients who visited Nayoro City General Hospital from November 2017 to December 2017, for comparison with the ripasudil group. The inclusion criteria for the control group were the presence of DME (FT at baseline ≧340 µm, which differed from the ripasudil group for adjusting the baseline FT between the groups), no history of any change in ophthalmic drugs, no history of ocular surgery (including laser surgery), and/or no treatment for macular edema such as injection of an intravitreal anti-VEGF drug within the previous 3 months and during the 2 to 12 weeks until the next examination. The patients did not receive any therapies for DME because of the risk of complications, financial reasons, or their own personal reasons. We enrolled 14 eyes of 10 patients as the control group in the study.

All patients underwent ophthalmologic examinations at baseline and at the first follow-up visit, including measurement of the best-corrected visual acuity (BCVA) and IOP, slit-lamp biomicroscopy with a noncontact fundus lens, and spectral-domain optical coherence tomography (SD-OCT) (RetinaScan RS-3000, Nidek, Gamagori, Japan). The BCVA was measured using a standard Japanese decimal visual acuity (VA) chart at 5 meters. The decimal values were converted to the logarithm of the minimum angle of resolution (logMAR) units for statistical analyses. To evaluate the FT, the macular map analysis protocol of the RS-3000 SD-OCT was used. The FT was defined as the average of all points in the inner circle (radius of 1 millimeter) at the center of the nine sectors defined by the Early Treatment Diabetic Retinopathy Study grid^17^.

### Data analysis

All values are expressed as the mean ± standard deviation. The differences in the FT, logMAR VA, and IOP between baseline and the first follow-up visit were assessed using the Wilcoxon signed-rank test. The differences between the study groups in the baseline FT, baseline logMAR VA, baseline IOP, changes in FT, changes in logMAR VA, and changes in IOP were assessed using a generalized linear mixed model (GLMM). The differences in age, duration of diabetes, and hemoglobin A1c (HbA1c) level between the study groups were assessed using the Mann-Whitney U test. *P* < 0.05 was considered significant.

## Results

Table 1 shows the baseline characteristics of all patients.

**Table 1:** Baseline Patient Characteristics.

Subgroup	Ripasudil	Control	*P* value
Patients (eyes)	10 (12)	10 (14)	
Age (years), mean ± SD	61.8 ± 20.7	70.2 ± 13.6	0.44
Gender (male/female)	4/6	7/3	
Duration of diabetes (years), mean ± SD	13.1 ± 8.4	16.1 ± 9.2	0.50
History of photocoagulation (eyes)	9 (75%)	7 (50%)	
HbA1c (%), mean ± SD	7.6 ± 1.7	6.9 ± 1.1	0.46
Lens status (phakic/pseudophakic)	6/6	8/6	
BCVA (logMAR), mean ± SD	0.39 ± 0.26	0.29 ± 0.37	0.77
(Snellen equivalent)	20/49	20/38	
Baseline FT (µm), mean ± SD	439 ± 72	402 ± 54	0.59
Baseline IOP (mmHg), mean ± SD	17.3 ± 5.2	11.7 ± 2.9	0.22

SD, standard deviation; HbA1c, hemoglobin A1c; BCVA, best-corrected visual acuity; FT, foveal thickness; logMAR, logarithm of the minimum angle of resolution; IOP, intraocular pressure.

All patients had type 2 diabetes mellitus. Eleven eyes had not received any other eye drops to treat POAG or OH before prescription of ripasudil. Latanoprost 0.005% with timolol 0.50% (Xalacom®, Pfizer, Tokyo, Japan) had been administered to treat glaucoma in one of the 12 study eyes before ripasudil was added. There were no significant differences between the groups with respect to baseline FT (439 ± 72 µm vs. 402 ± 54 µm), baseline logMAR VA (0.39 ± 0.26 vs. 0.29 ± 0.37, 20/49 vs. 20/38 Snellen equivalent), baseline IOP (17.3 ± 5.2 mmHg vs. 11.7 ± 2.9 mmHg), duration of diabetes, or HbA1c level.

In the ripasudil group, the mean FT decreased significantly (*P* = 0.003) from 439 ± 72 µm at baseline to 395 ± 62 µm at 1 month. The reduction in the mean FT from baseline to 1 month in the ripasudil group (−44 ± 42 µm) was significantly (*P* = 0.01) greater than that in the control group (1 ± 39 µm). The mean IOP decreased significantly (*P* = 0.02) from 17.3 ± 5.2 mmHg at baseline to 14.6 ± 4.0 mmHg at 1 month. The reduction in the mean IOP from baseline to 1 month in the ripasudil group (−2.7 ± 2.9 mmHg) was significantly (*P* = 0.008) greater than that in the control group (0 ± 1.6 mmHg). There was no significant change in the logMAR VA in the ripasudil group from baseline to 1 month (0.39 ± 0.26 to 0.38 ± 0.22, 20/49 to 20/48 Snellen equivalent,). There was no significant difference in the change in logMAR VA (−0.01 ± 0.1 vs. 0.03 ± 0.1) between the ripasudil and control groups. Table 2 shows the changes in each parameter in the study groups.

**Table 2:** Changes in Each Parameter in the Study Groups.

Group (eyes)	Ripasudil (12)	Control (14)
FT (µm) mean ± SD	−44 ± 42*	1 ± 39
IOP (mmHg) mean ± SD	−2.7 ± 2.9*	0 ± 1.6
BCVA (logMAR) mean ± SD	−0.01 ± 0.1	0.03 ± 0.1

*P < 0.05, generalized linear mixed model.

SD, standard deviation; IOP, intraocular pressure; FT, foveal thickness; BCVA, best-corrected visual acuity; logMAR, logarithm of the minimum angle of resolution.

## Discussion

In the current study, we showed that the IOP and FT decreased significantly at approximately 1 month (range, 2 to 8 weeks) after the initiation of ripasudil therapy, suggesting that ripasudil might effectively reduce IOP and improve DME, although the VA did not improve following therapy.

Previous reports have shown that ROCK inhibitors alter the cellular components of the trabecular meshwork and Schlemm’s canal cells in the outflow pathway of the aqueous humor, decreasing the outflow resistance and reducing the IOP^11,18^. ROCK is also involved in angiogenesis, hyperpermeability, and the pathogenesis of various pathologies, such as inflammation and fibrosis. Some studies have reported that a ROCK inhibitor is beneficial for retinal diseases, including DR and DME^12–16,19–24^. Hida *et al*. reported the effects of ripasudil on retinal edema and nonperfusion areas in a murine model of retinal vein occlusion^25^. Nourinia *et al*. and Ahmadieh *et al*. showed that a combination therapy of bevacizumab and a ROCK inhibitor (fasudil) in an intravitreal injection was effective in eyes with severe DME that were refractory to anti-VEGF therapy^15,16^. In addition, Isobe *et al*. reported that when a radiolabeled drug was used, ripasudil reached the retina and choroid after the administration of eye drops in rabbits^26^. Therefore, we considered that ripasudil might have the potential to reduce the FT in patients with DME. Although the mechanism of reduction in DME after the administration of ripasudil remains unclear, Hida *et al*. suggested that ripasudil reduces macular edema by regulating the tight junction integrity in the retina^25^. In addition, Nourinia *et al*. and Ahmadieh *et al*. suggested that a ROCK inhibitor causes positive effects on DME by directly protecting vascular endothelial cells^15,16^.

Anti-VEGF agents have become the first-line treatment for DME. However, the treatment requires repeated intravitreous injections for an indefinite period, and the treatment cost is a significant burden on patients^27^. Although anti-VEGF drugs have low rates of adverse events, the topical and/or systemic adverse events such as endophthalmitis and cerebrovascular accidents are serious^5,6,8,28^. Furthermore, a large percentage of patients still have a poor response to anti-VEGF agents even with frequently repeated injections^5,8^. Although intravitreous injections of steroids are also effective for treating DME, the injections are associated with more frequent ocular side effects, such as the development of cataracts and glaucoma, compared to intravitreous injections of anti-VEGF agents^29^. Therefore, other therapeutic modalities with different mechanisms of action should be developed to overcome these side effects and the shortcomings of anti-VEGF and steroid injections, i.e., more cost-effective and safer treatments are required. Previous reports have shown that combination therapy of bevacizumab and fasudil intravitreal injections improves DME in eyes refractory to currently available anti-VEGF therapy, indicating that ROCK inhibition has a mechanism that differs from that of anti-VEGF therapy^15,16^. For those reasons, the results of the current study might be useful to develop a new treatment modality for DME.

The current study showed that the BCVA did not improve despite reductions in the FT. A previous clinical trial of laser therapy for DME reported that BCVA improvements from baseline were correlated modestly with reductions in the FT from baseline^30^. However, there are substantial variations in the BCVA levels at any given FT^31^. The current results showed no significant correlation between the BCVA improvements from baseline and reductions in the FT from baseline. In the current study, the degree of reduction in the FT was smaller than those reported previously with anti-VEGF therapy, and the reassessment period was short^4–8^. Furthermore, some reports have indicated that the improvement in BCVA after anti-VEGF therapy is correlated with the baseline BCVA, systemic factors, and OCT characteristics^4,5,32–35^. The current study could not determine the effects of those factors on the discrepancy between the reductions in FT and BCVA improvements. Prospective clinical studies with a long follow-up period and more participants should determine whether ripasudil improves BCVA in patients with DME.

The current study had some limitations. First, the number of patients was too small to facilitate subgroup analysis, although it is difficult to recruit subjects with these specific conditions. Although the current results showed that the efficacy of ripasudil varied among patients (the changes in the FT values ranged from −143 to 0 µm), we did not determine the characteristic differences between the patients who had improvements in FT and those who did not. Another clinical study that includes more patients and subgroup analyses regarding the differences in response to ripasudil is needed. Second, the current follow-up period was short. Third, the current study was retrospective, which introduced potential biases. Prospective, long-term studies that include more patients with DME treated with ripasudil are needed.

In conclusion, the current findings showed that IOP and FT were decreased significantly at 1 month (range, 2 to 8 weeks) after the initiation of ripasudil therapy, suggesting that ripasudil has the potential to improve IOP and FT in patients with DME.

### Ethics approval and consent to participate

The Ethics Committee of Nayoro City General Hospital approved this study.

Nayoro City General Hospital Ethics Committee. Reference number 111-7.

## Data Availability

The datasets procured and/or analyzed during the current study are available from the corresponding author on reasonable request.
